# *N*′-(2-Hy­droxy­benzyl­idene)-2-(hy­droxy­imino)­propano­hydrazide

**DOI:** 10.1107/S1600536811045818

**Published:** 2011-11-12

**Authors:** Maxym O. Plutenko, Rostyslav D. Lampeka, Yurii S. Moroz, Matti Haukka, Svetlana V. Pavlova

**Affiliations:** a Department of Chemistry, National Taras Shevchenko University, Volodymyrska Street 64, 01601 Kyiv, Ukraine; b Department of Chemistry, University of Joensuu, PO Box 111, 80101 Joensuu, Finland

## Abstract

The mol­ecule of the title compound, C_10_H_11_N_3_O_3_, adopts an all-*trans* conformation and is approxomately planar, the largest deviation from the least-squares plane through all non-H atoms being 0.261 (1) Å. An intra­molecular O—H⋯N hydrogen bond occurs. In the crystal, the mol­ecules are packed into layers lying parallel to the *ab* plane by π-stacking inter­actions between the benzene ring of one molecule and the C—N bond of the oxime group of another molecule; the shortest inter­molecular C⋯C separation within the layer is 3.412 (1) Å. The layers are connected by O—H⋯O and N—H⋯O hydrogen bonds.

## Related literature

For the preparation and characterization of 3*d* metal complexes with related oxime derivatives, see: Kanderal *et al.* (2005 ▶); Moroz *et al.* (2010 ▶). For the crystal structures of similar oxime derivatives, see: Świątek-Kozłowska *et al.* (2000 ▶); Mokhir *et al.* (2002 ▶); Sachse *et al.* (2008 ▶). For 2-hy­droxy­imino­propanamide and amide derivatives of 2-hy­droxy­imino­propanoic acid, see: Onindo *et al.* (1995 ▶); Duda *et al.* (1997 ▶); Sliva *et al.* (1997 ▶). For the synthesis of 2-(hy­droxy­imino)­propane­hydrazide, see: Fritsky *et al.* (1998 ▶). For related structures, see: Krämer & Fritsky (2000 ▶); Wörl *et al.* (2005 ▶).
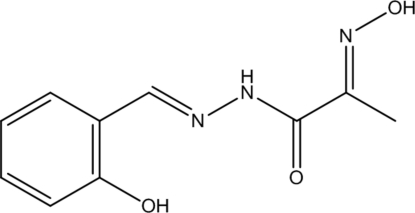


## Experimental

### 

#### Crystal data

C_10_H_11_N_3_O_3_
*M* *_r_* = 221.22
Monoclinic, 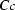

*a* = 11.2296 (4) Å
*b* = 8.1905 (4) Å
*c* = 11.1000 (5) Å
β = 102.223 (2)°
*V* = 997.79 (8) Å^3^
*Z* = 4
Mo *K*α radiation
μ = 0.11 mm^−1^
*T* = 100 K
0.61 × 0.47 × 0.34 mm

#### Data collection

Bruker Kappa APEXII DUO CCD diffractometer
Absorption correction: multi-scan (*SADABS*; Sheldrick, 2008 ▶) *T*_min_ = 0.935, *T*_max_ = 0.964
16456 measured reflections
2465 independent reflections
2383 reflections with *I* > 2σ(*I*)
*R*_int_ = 0.015

#### Refinement

*R*[*F* ^2^ > 2σ(*F* ^2^)] = 0.029
*wR*(*F* ^2^) = 0.086
*S* = 1.07
2465 reflections
148 parameters
2 restraints
H-atom parameters constrained
Δρ_max_ = 0.42 e Å^−3^
Δρ_min_ = −0.22 e Å^−3^

### 

Data collection: *APEX2* (Bruker, 2010 ▶); cell refinement: *SAINT* (Bruker, 2009 ▶); data reduction: *SAINT*; program(s) used to solve structure: *SHELXS97* (Sheldrick, 2008 ▶); program(s) used to refine structure: *SHELXL97* (Sheldrick, 2008 ▶); molecular graphics: *DIAMOND* (Brandenburg, 2011 ▶); software used to prepare material for publication: *SHELXL97*.

## Supplementary Material

Crystal structure: contains datablock(s) I, global. DOI: 10.1107/S1600536811045818/yk2025sup1.cif

Structure factors: contains datablock(s) I. DOI: 10.1107/S1600536811045818/yk2025Isup2.hkl

Supplementary material file. DOI: 10.1107/S1600536811045818/yk2025Isup3.mol

Supplementary material file. DOI: 10.1107/S1600536811045818/yk2025Isup4.cml

Additional supplementary materials:  crystallographic information; 3D view; checkCIF report

## Figures and Tables

**Table 1:** Hydrogen-bond geometry (Å, °)

*D*—H⋯*A*	*D*—H	H⋯*A*	*D*⋯*A*	*D*—H⋯*A*
O1—H1⋯N1	0.84	1.85	2.5808 (10)	144
O3—H3⋯O2^i^	0.84	1.82	2.6518 (9)	171
N2—H2⋯O1^ii^	0.88	2.32	3.1535 (9)	157

Symmetry codes: (i) 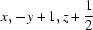; (ii) 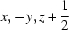.

**Fig. 1.**
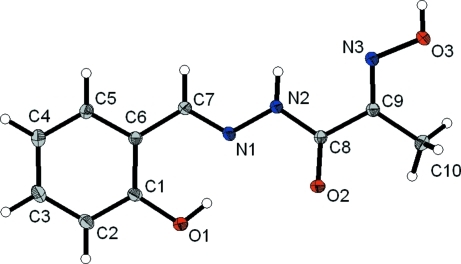
The molecular structure of the title compound, with displacement ellipsoids shown at the 50% probability level and atom labelling.

**Fig. 2.**
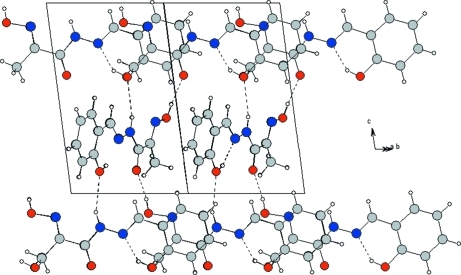
Crystal packing of the title compound.

## supplementary crystallographic information

### Comment

Polynucleative oxime ligands attract considerable interest because of their ability to act as efficient chelating agents with respect to 3 d-metal ions and their tendency to form polynuclear metal complexes (Kanderal *et al.* 2005; Moroz *et al.* 2010). In the present work we present the synthesis and structure of the title compound (1) (Fig. 1), which comprises several donor groups: oxime, hydrazone, azomethine, and phenolic.

In the structure 1 the *N*'-(2-hydroxybenzylidene)-2-(hydroxyimino)propanehydrazide molecules are connected by extensive system of hydrogen bonds. The bond lengths N—O and C—N in the oxime group are 1.3838 (9) and 1.2854 (9) Å respectively, which is typical for protonated moieties of this type (Świątek-Kozłowska *et al.*, 2000; Mokhir *et al.*, 2002; Sachse *et al.*, 2008). The oxime group is in a *trans* position with respect to the amide group, in accordance with the structures of 2-hydroxyiminopropanamide and other amide derivatives of 2-hydroxyiminopropanoic acid (Onindo *et al.*, 1995; Duda *et al.*, 1997; Sliva *et al.*, 1997).

Bond lengths N—N', N—C and C—O of the hydrazone group are 1.3643 (9), 1.3544 (9) and 1.2353 (9) Å, respectively, and are typical for the protonated hydrazone groups (Moroz *et al.*, 2010). The oxime and the hydrazide groups are situated in *trans*-position around the C(8)—C(9) bond. The CH_3_C(NOH)C(O)NH fragment is almost planar (deviations of the non-hydrogen atoms from the moiety's mean plane are less than 0.2 Å).

The C—C (1.3814 (11) – 1.4115 (9) Å) bond lengths in the benzene ring have their typical values (Krämer *et al.*, 2000; Wörl *et al.*, 2005). The angles C—C'-C'', C—N—C' and N—C—C' are near 120°.

There are three hydrogen bonds in structure of 1 (Table 2). The O1—H1···N1 is an intramolecular hydrogen bond, where the phenolic oxygen atom acts as donor and the azomethine nitrogen atom acts as receptor. The O3—H3···O2 and N2—H2···O1 hydrogen bonds are intermolecular, the oximic oxygen and the hydrazone nitrogen atoms act as donors and the hydrazone oxygen and the phenolic oxygen atoms act as acceptors.

In the crystal packing, molecules of 1 form layers parallel to *ab* plane. The molecules in the layer are connected by π-stacking between the benzene ring of one molecule and C—N bond of the oxime group of another molecule. The distance between two planes formed by neighboring molecules is 3.3493 (7) Å. The layers are connected by extensive system of hydrogen bonds.

### Experimental

A mixture of 2-(hydroxyimino)propanehydrazide synthesized according to (Fritsky *et al.*, 1998) (0.117 g, 0.1 mmol) and salicylic aldehyde (0.122 g, 0.1 mmol) in 10 ml of methanol was heated to reflux for 2 h. On cooling to room temperature, a solid precipitate was formed. The solid was filtered and then recrystallized from methanol. Yellowish needle crystals of 1 were obtained by slow evaporation of the methanolic solution. Yield: 2 g (90%).

### Refinement

All hydrogen atoms were positioned geometrically and constrained to ride on their parent atoms, with C—H = 0.95–0.98 Å, N—H = 0.88 Å, O—H = 0.84 Å, and *U*_iso_ = 1.2–1.5 *U*_eq_(parent atom). The highest peak is located 0.64 Å from atom N3 and the deepest hole is located 0.30 Å from atom H3.

### Crystal data

**Table:**

C_10_H_11_N_3_O_3_	*F*(000) = 464
*M**_r_* = 221.22	*D*_x_ = 1.473 Mg m^−^^3^
Monoclinic, *C**c*	Mo *K*α radiation, λ = 0.71073 Å
Hall symbol: C -2yc	Cell parameters from 9875 reflections
*a* = 11.2296 (4) Å	θ = 3.1–36.5°
*b* = 8.1905 (4) Å	µ = 0.11 mm^−^^1^
*c* = 11.1000 (5) Å	*T* = 100 K
β = 102.223 (2)°	Block, yellow
*V* = 997.79 (8) Å^3^	0.61 × 0.47 × 0.34 mm
*Z* = 4	

### Data collection

**Table:**

Bruker Kappa APEXII DUO CCD diffractometer	2465 independent reflections
Radiation source: fine-focus sealed tube	2383 reflections with *I* > 2σ(*I*)
curved graphite crystal	*R*_int_ = 0.015
Detector resolution: 16 pixels mm^-1^	θ_max_ = 36.6°, θ_min_ = 3.1°
φ scans and ω scans with κ offset	*h* = −18→18
Absorption correction: multi-scan (*SADABS*; Sheldrick, 2008)	*k* = −13→13
*T*_min_ = 0.935, *T*_max_ = 0.964	*l* = −17→18
16456 measured reflections	

### Refinement

**Table:**

Refinement on *F*^2^	Primary atom site location: structure-invariant direct methods
Least-squares matrix: full	Secondary atom site location: difference Fourier map
*R*[*F*^2^ > 2σ(*F*^2^)] = 0.029	Hydrogen site location: inferred from neighbouring sites
*wR*(*F*^2^) = 0.086	H-atom parameters constrained
*S* = 1.07	*w* = 1/[σ^2^(*F*_o_^2^) + (0.0648*P*)^2^ + 0.0678*P*] where *P* = (*F*_o_^2^ + 2*F*_c_^2^)/3
2465 reflections	(Δ/σ)_max_ < 0.001
148 parameters	Δρ_max_ = 0.42 e Å^−^^3^
2 restraints	Δρ_min_ = −0.22 e Å^−^^3^

### Special details

**Table:**

Geometry. All s.u.'s (except the s.u.'s in the dihedral angle between two l.s. planes) are estimated using the full covariance matrix. The cell s.u.'s are taken into account individually in the estimation of s.u.'s in distances, angles and torsion angles; correlations between s.u.'s in cell parameters are only used when they are defined by crystal symmetry. An approximate (isotropic) treatment of cell s.u.'s is used for estimating s.u.'s involving l.s. planes.
Refinement. Refinement of *F*^2^ against ALL reflections. The weighted *R*-factor *wR* and goodness of fit *S* are based on *F*^2^, conventional *R*-factors *R* are based on *F*, with *F* set to zero for negative *F*^2^. The threshold expression of *F*^2^ > σ(*F*^2^) is used only for calculating *R*-factors(gt) *etc*. and is not relevant to the choice of reflections for refinement. *R*-factors based on *F*^2^ are statistically about twice as large as those based on *F*, and *R*- factors based on ALL data will be even larger.

### Fractional atomic coordinates and isotropic or equivalent isotropic displacement parameters (Å^2^)

**Table:**

	*x*	*y*	*z*	*U*_iso_*/*U*_eq_	
O1	0.43931 (7)	−0.17009 (9)	0.11307 (6)	0.01652 (13)	
H1	0.4154	−0.0863	0.1446	0.025*	
O2	0.34012 (7)	0.24663 (9)	0.12079 (6)	0.01761 (13)	
O3	0.27014 (7)	0.63005 (9)	0.39602 (7)	0.01675 (13)	
H3	0.2975	0.6608	0.4688	0.025*	
N1	0.43375 (7)	0.03499 (9)	0.28832 (7)	0.01324 (13)	
N2	0.39288 (7)	0.18082 (9)	0.32287 (6)	0.01328 (12)	
H2	0.3983	0.2071	0.4007	0.016*	
N3	0.32114 (7)	0.48105 (9)	0.37696 (7)	0.01365 (13)	
C1	0.50374 (8)	−0.26684 (11)	0.20397 (8)	0.01297 (13)	
C2	0.54689 (9)	−0.41516 (12)	0.16937 (8)	0.01686 (15)	
H2A	0.5302	−0.4465	0.0851	0.020*	
C3	0.61440 (9)	−0.51730 (12)	0.25817 (9)	0.01803 (16)	
H3A	0.6450	−0.6173	0.2337	0.022*	
C4	0.63779 (9)	−0.47511 (11)	0.38250 (9)	0.01695 (15)	
H4	0.6840	−0.5455	0.4427	0.020*	
C5	0.59273 (8)	−0.32933 (11)	0.41698 (8)	0.01476 (14)	
H5	0.6073	−0.3011	0.5019	0.018*	
C6	0.52600 (8)	−0.22227 (10)	0.32956 (7)	0.01217 (13)	
C7	0.48236 (8)	−0.06904 (11)	0.37043 (7)	0.01343 (14)	
H7	0.4897	−0.0466	0.4557	0.016*	
C8	0.34332 (8)	0.28258 (10)	0.22951 (7)	0.01258 (13)	
C9	0.29200 (7)	0.43934 (10)	0.26290 (7)	0.01256 (13)	
C10	0.21336 (9)	0.53605 (12)	0.16288 (8)	0.01674 (15)	
H10A	0.1476	0.5877	0.1948	0.025*	
H10B	0.1784	0.4635	0.0943	0.025*	
H10C	0.2625	0.6204	0.1339	0.025*	

### Atomic displacement parameters (Å^2^)

**Table:**

	*U*^11^	*U*^22^	*U*^33^	*U*^12^	*U*^13^	*U*^23^
O1	0.0240 (3)	0.0136 (3)	0.0116 (2)	0.0019 (2)	0.0029 (2)	0.0001 (2)
O2	0.0277 (3)	0.0146 (3)	0.0108 (2)	0.0026 (2)	0.0044 (2)	−0.0002 (2)
O3	0.0226 (3)	0.0130 (3)	0.0146 (2)	0.0040 (2)	0.0037 (2)	−0.0023 (2)
N1	0.0175 (3)	0.0093 (3)	0.0132 (3)	0.0009 (2)	0.0040 (2)	−0.0002 (2)
N2	0.0185 (3)	0.0099 (3)	0.0115 (2)	0.0019 (2)	0.0034 (2)	−0.0002 (2)
N3	0.0167 (3)	0.0111 (3)	0.0132 (3)	0.0007 (2)	0.0034 (2)	−0.0006 (2)
C1	0.0156 (3)	0.0117 (3)	0.0121 (3)	−0.0010 (2)	0.0040 (2)	−0.0008 (2)
C2	0.0217 (4)	0.0137 (3)	0.0161 (3)	0.0011 (3)	0.0062 (3)	−0.0024 (3)
C3	0.0201 (4)	0.0137 (3)	0.0215 (4)	0.0026 (3)	0.0071 (3)	−0.0018 (3)
C4	0.0176 (4)	0.0136 (3)	0.0198 (3)	0.0023 (3)	0.0042 (3)	0.0016 (3)
C5	0.0169 (3)	0.0125 (3)	0.0142 (3)	0.0007 (3)	0.0019 (3)	0.0011 (2)
C6	0.0149 (3)	0.0102 (3)	0.0115 (3)	−0.0006 (2)	0.0030 (2)	0.0002 (2)
C7	0.0169 (3)	0.0113 (3)	0.0117 (3)	0.0007 (3)	0.0023 (2)	−0.0006 (2)
C8	0.0155 (3)	0.0102 (3)	0.0121 (3)	−0.0001 (2)	0.0028 (2)	0.0004 (2)
C9	0.0145 (3)	0.0109 (3)	0.0123 (3)	0.0000 (2)	0.0029 (2)	0.0001 (2)
C10	0.0178 (4)	0.0173 (4)	0.0145 (3)	0.0039 (3)	0.0019 (3)	0.0022 (3)

### Geometric parameters (Å, °)

**Table:**

O1—C1	1.3640 (11)	C3—C4	1.3926 (14)
O1—H1	0.8400	C3—H3A	0.9500
O2—C8	1.2352 (10)	C4—C5	1.3822 (13)
O3—N3	1.3832 (10)	C4—H4	0.9500
O3—H3	0.8400	C5—C6	1.4014 (11)
N1—C7	1.2822 (10)	C5—H5	0.9500
N1—N2	1.3634 (10)	C6—C7	1.4543 (12)
N2—C8	1.3543 (10)	C7—H7	0.9500
N2—H2	0.8800	C8—C9	1.4861 (12)
N3—C9	1.2850 (11)	C9—C10	1.4916 (12)
C1—C2	1.3917 (12)	C10—H10A	0.9800
C1—C6	1.4113 (11)	C10—H10B	0.9800
C2—C3	1.3891 (14)	C10—H10C	0.9800
C2—H2A	0.9500		
			
C1—O1—H1	109.5	C4—C5—H5	119.3
N3—O3—H3	109.5	C6—C5—H5	119.3
C7—N1—N2	120.02 (7)	C5—C6—C1	118.62 (7)
C8—N2—N1	115.60 (7)	C5—C6—C7	119.34 (7)
C8—N2—H2	122.2	C1—C6—C7	122.03 (7)
N1—N2—H2	122.2	N1—C7—C6	118.21 (7)
C9—N3—O3	110.98 (7)	N1—C7—H7	120.9
O1—C1—C2	117.65 (7)	C6—C7—H7	120.9
O1—C1—C6	122.40 (7)	O2—C8—N2	121.54 (8)
C2—C1—C6	119.95 (8)	O2—C8—C9	121.13 (7)
C3—C2—C1	119.97 (8)	N2—C8—C9	117.32 (7)
C3—C2—H2A	120.0	N3—C9—C8	116.37 (7)
C1—C2—H2A	120.0	N3—C9—C10	125.40 (8)
C2—C3—C4	120.92 (9)	C8—C9—C10	118.22 (7)
C2—C3—H3A	119.5	C9—C10—H10A	109.5
C4—C3—H3A	119.5	C9—C10—H10B	109.5
C5—C4—C3	119.05 (8)	H10A—C10—H10B	109.5
C5—C4—H4	120.5	C9—C10—H10C	109.5
C3—C4—H4	120.5	H10A—C10—H10C	109.5
C4—C5—C6	121.48 (8)	H10B—C10—H10C	109.5

### Hydrogen-bond geometry (Å, °)

**Table:**

*D*—H···*A*	*D*—H	H···*A*	*D*···*A*	*D*—H···*A*
O1—H1···N1	0.84	1.85	2.5808 (10)	144.
O3—H3···O2^i^	0.84	1.82	2.6518 (9)	171.
N2—H2···O1^ii^	0.88	2.32	3.1535 (9)	157.

Symmetry codes: (i) *x*, −*y*+1, *z*+1/2; (ii) *x*, −*y*, *z*+1/2.
